# Exploring the Potential of HySpex Hyperspectral Imagery for Extraction of Copper Content

**DOI:** 10.3390/s20216325

**Published:** 2020-11-06

**Authors:** Guo Jiang, Shuguang Zhou, Shichao Cui, Tao Chen, Jinlin Wang, Xi Chen, Shibin Liao, Kefa Zhou

**Affiliations:** 1 State Key Laboratory of Desert and Oasis Ecology, Xinjiang Institute of Ecology and Geography, Chinese Academy of Sciences, Urumqi 830011, China; jiangguo16@mails.ucas.ac.cn (G.J.); zhoushuguang@ms.xjb.ac.cn (S.Z.); cuishichao14@mails.ucas.edu.cn (S.C.); wangjinlin@ms.xjb.ac.cn (J.W.); chenxi176@mails.ucas.edu.cn (X.C.); liaoshibin@ms.xjb.ac.cn (S.L.); 2 Xinjiang Key Laboratory of Mineral Resources and Digital Geology, Urumqi 830011, China; 3 Research Centre for Mineral Resources, Chinese Academy of Sciences, Urumqi 830011, China; 4 University of Chinese Academy of Sciences, Beijing 100049, China; 5 Department of Physical Geography, Resources and Environment, School of Geography and Planning, Sun Yat-sen University, Guangzhou 510275, China; chent265@mail2.sysu.edu.cn

**Keywords:** HySpex, spectra transform, wavebands selection, partial least square (PLS), geochemistry

## Abstract

Detritus geochemical information has been proven through research to be an effective prospecting method in mineral exploration. However, the traditional detritus metal content monitoring methods based on field sampling and laboratory chemical analysis are time-consuming and may not meet the requirements of large-scale metal content monitoring. In this study, we obtained 95 detritus samples and seven HySpex hyperspectral imagery scenes with a spatial resolution of 1 m from Karatag Gobi area, Xinjiang, China, and used partial least squares and wavebands selection methods to explore the usefulness of super-low-altitude HySpex hyperspectral images in estimating detritus feasibility and effectiveness of Cu element content. The results show that: (1) among all the inversion models of transformed spectra, power-logarithm transformation spectrum was the optimal prediction model (coefficient of determination(*R*^2^) = 0.586, mean absolute error(MAE) = 21.405); (2) compared to the genetic algorithm (GA) and continuous projection algorithm (SPA), the competitive weighted resampling algorithm (CARS) was the optimal feature band-screening method. The *R*^2^ of the inversion model was constructed based on the characteristic bands selected by CARS reaching 0.734, which was higher than that of GA (0.519) and SPA (0.691), and the MAE (19.926) was the lowest. Only 20 bands were used in the model construction, which is lower than that of GA (105) and SPA (42); (3) The power-logarithm transforms, and CARS combined with the model of HySpex hyperspectral images and the Cu content distribution in the study area were obtained, consistent with the actual survey results on the ground. Our results prove that the method incorporating the HySpex hyperspectral data to invert copper content in detritus is feasible and effective, and provides data and a reference method for obtaining geochemical element distribution in a large area and for reducing key areas of geological exploration in the future.

## 1. Introduction

Detritus are mainly derived from the differentiation of ore bodies and surrounding rocks [1]. Detritus geochemical measurement is a geochemical survey method used to study the enrichment and dispersion of elements by collecting detritus for geochemical research and mineral resource exploration. The bedrock outcrops are scarce in the Gobi region of eastern Xinjiang, China, and the quaternary cover is widely distributed. Studies showed that detritus are developed in the region, thus the use of the detritus geochemical survey method for mineral exploration can produce better results than water system sediment geochemical surveys [2,3,4]. Therefore, the rapid and effective acquisition of content of various elements in detritus samples is important for detailed mineral exploration of potential metallogenic areas.

The traditional detritus geochemical survey method usually involves collecting detritus samples according to the grid method, and then obtaining the element contents of the samples in the laboratory by chemical analysis or element detector. The final step is obtaining the spatial distribution of metal elements using the interpolation method, such as kriging. [5,6]. Although this method produces results, it is both time-consuming and expensive and cannot quickly obtain a wide range of element content distribution information. Human, material, and financial resources are needed in areas with harsh geological conditions [7,8]. Conversely, remote sensing can cover large areas with high efficiency and low cost, which is theoretically significant and has application value for discovering mineralization anomalies and shrinking detailed investigation area, as well as improving exploration efficiency and accelerating the process of mineral exploration [9].

Research results in the past few decades have shown that the spectrum in the visible-near-infrared range is mainly affected by the electronic transition of metal cations, but the spectrum in the short-wave infrared range is mainly affected by the oscillation of anionic groups [10,11]. The size and content of cations reflect the spectral reflectance. The increase in aluminum content causes the absorption valley to shift from right to left, whereas Cu, Zn, Ni, Cd, As, Cr, Ni, and Pb, etc., elements are correlated with the physical and chemical properties of soil (organic matter, clay minerals, and soil pH) [12,13,14]. Theabove-mentioned theories proved the feasibility of the use the remote sensing technology to retrieve geochemical element content.

To date, remote sensing has been successfully used to retrieve metal element contents using methods such as linear analysis (partial least squares, ridge regression, stepwise regression, etc.) and nonlinear mathematical analysis (random forest, extreme learning machine, support vector machine, etc.) and to establish the quantitative relationship between metal element content and spectral reflectance [15,16,17,18,19]. These are point models based on the spectral reflectance and element content of samples obtainied from field sites or the laboratory. Although the model constructed by this method has high prediction accuracy, it can only provide field information. The spatial distribution of element content was obtained by spatial interpolation because the spatial heterogeneity is usually different to the actual results [20].

In recent years, with the development of imaging spectroscopy technology, the spectral and spatial resolutions of sensors have been greatly improved; scholars began to adopt the hyperspectral images to directly capture surface metal content inversion. The imaging spectrum not only acquires traditional point spectrum information in space, but also spatially continuous image information. Many previous studies directly applied the laboratory established point model to the pixel spectrum [21,22,23]. Due to the physicochemical properties (moisture, surface roughness, organic matter content, etc.) and the limited spatial resolution of aerospace remote sensing images, the direct application of the point model established on the ground may lead to uncertainty in the results [24,25,26,27]. With the development of simple and stable airborne platform technology, low-altitude imaging hyperspectral remote sensing has received wide attention [28,29]. Influential factors such as atmospheric interference, a large amount of data, and spectral noise may reduce the accuracy of the model and weaken the robustness of the model [30,31]. It is necessary to study the applicable spectral pretreatment technology and characteristic band selection methods to improve the performance of the models based on aerial hyperspectral images [32,33].

To solve the above problems, we discussed whether a correspondence exists between the spectral reflectance of image pixels and the measured Cu content on the ground. We determined the validity of Cu element content estimation using the image pixel spectrum obtained by an ultra-low-altitude detection platform. This study was based on the power delta wing and HySpex imaging spectrometer to form an “ultra-low altitude hyperspectral detection platform”. According to the research requirements, visible-near-infrared hyperspectral images with a spatial resolution of 1 m and 216 bands were obtained in the study area. We simultaneously collected 95 detritus samples (1 × 1 m). We established a quantitative model between the pixel spectrum and element content by combining spectral transform technology and a characteristic band selection algorithm, then we verified the accuracy and practicability of the proposed method based on field works. This method can be used to improve prospecting efficiency and expand the prospecting space in the prediction of element content.

## 2. Materials and Methods

### 2.1. Study Area

**Figure 1:**
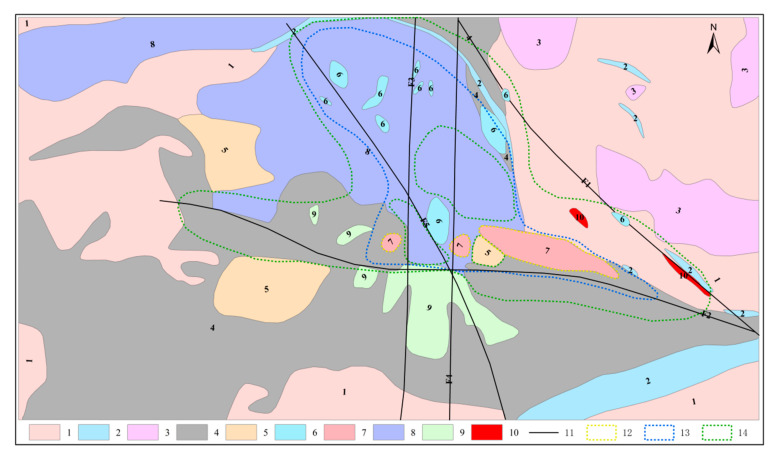
Geological map of the study area (after Mao et al., 2017) [34], 1. Clastic sedimentary rock; 2. biogenic carbonates; 3. volcainc breccia; 4. dacitic volcanic and volcaniclastic rocks; 5. basalt; 6. pyrite felsite; 7. mineralized quartz diorite porphyry; 8. diorite porphyry; 9. Gabbro intrusion; 10. siderite ore bodies; 11. faults; 12. potassic + silication zone; 13. silication + sericitization zone; 14. propylitization zone.

**Figure 2:**
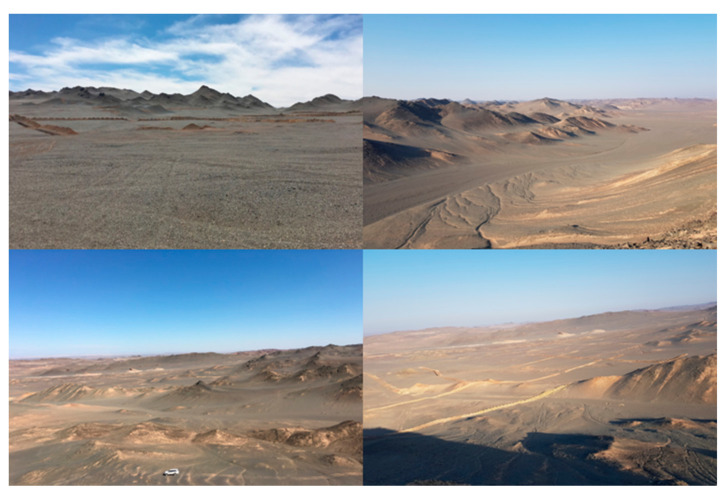
Local topography of the study area.

The Karatagh ore-centralization area is located in the southern margin of the Turpan-Hami basin of the East Tianshan metallogenic belt. Hongshan, Hongshi, and Meiling volcanic hydrothermal vein copper and gold deposits have been discovered in this area [34]. The study area is located in the northwest section of Karatagh uplift. The exposed strata in the south includes a set of basic-acid volcanic-pyroclastic rocks, and detrital sedimentary rocks of the Dananhu Formation in the north and east. The detrital sedimentary rocks unconformably overlaid on a set of pyroclastic rocks, in which lenticular hematite manganese ore and siderite bodies developed (Figure 1) [35]. Previous studies showed that there are more metallic elements anomalies in the region (Cu, Au, Ag, etc.) [36]. The surface is mainly composed of volcanic clastic rocks and andesite, with occasional copper mineralized rock outcrops which has the ore-forming geological conditions for the formation of porphyry copper gold deposits [35]. The region belongs to a typical continental semi-arid climate, with annual average precipitation and temperature of 34.9 mm and 38 °C, respectively, and an average altitude of about 500–600m [37]. There is no vegetation cover on the surface, and the surface rocks are severely weathered. The hills, which have a relative height difference of about several tens of meters are widely distributed. The whole landscape belongs to the hilly-quasi-plain desert-Gobi landform category (Figure 2). The geological outcrops are generally located in a relatively high terrain. Due to the low efficiency and the time-consuming and laborious exploration of mineral resources through traditional geochemical methods, coupled with the study area not being covered by vegetation, water bodies and buildings, remote-sensing technology in this research area is suitable for geochemical anomaly inversion research.

### 2.2. Data Acquisition and Preprocessing

**Figure 3:**
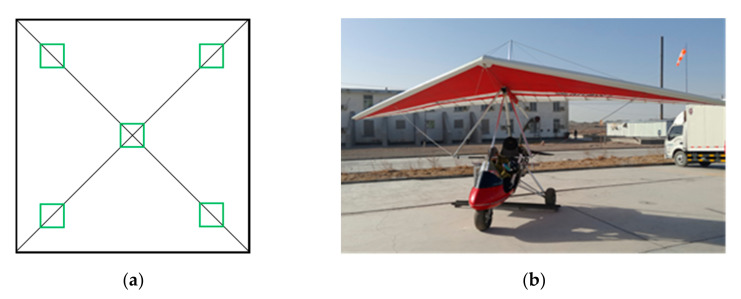
Sampling methods and hyperspectral image acquisition method: (**a**) schematic diagram of sampling quadrat; (**b**) aerial detection platform.

The data used in this study included HySpex hyperspectral images and detritus sample analysis data. The HySpex VNIR-1024 sensor is manufactured by Norsk Elektro Optikk A/S (NEO, Oslo, Norway), and its performance parameters are shown in Table 1. We obtained the hyperspectral images using the detection platform in September 2017, during clear weather and few thin clouds in the sky (Figure 3b). The HySpex hyperspectral image has a spatial resolution of 1 m, a spectral resolution of 2.7 nm, and a coverage area of 18 km^2^. Samples and image data were collected synchronously, and the sampling method was used to collect 3–5 detritus samples (the sample diameter was about 5 cm) in a range of 1 × 1m on the ground. We then placed them into a sample bag as a quadrate sample (Figure 3a). A portable handheld global positioning system (GPS) was used to record the coordinates of sampling points. All samples in this study were collected from 95 sampling points. After the samples were returned to the laboratory, a portable X-ray fluorescence spectrometer (pXRF); Niton XL3t 950, ThermoFisher Scientific (Niton), Boston, MA, USA) was used for analysis. In this study, the working mode of the instrument for sample analysis was set to the mineral (Cu/Zn) mode, and the measurement time of each sample was set to 120 s. We repeated the measurement three times, and the average value was taken as the sample content to ensure the accuracy and representativeness of element content analysis results.

**Figure 4:**
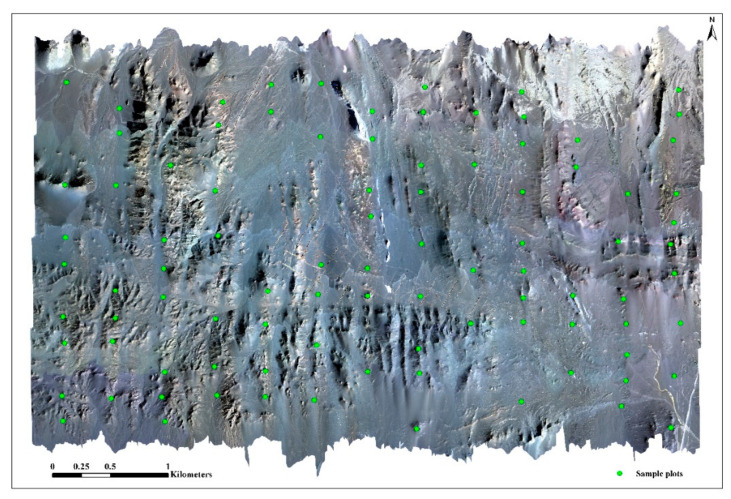
Map of the study region: Remote sensing image acquired using HySpex VNIR-1024 imaging sensors (Red: 641 nm; Green: 550 nm; Blue: 471 nm; spatial resolution: 1 m).

The image data collected in this paper can be used for further analysis after radiation calibration, atmospheric correction, and geometric correction [38]. HySpex RAD and HySpex NAV software (Norsk Elektro Optikk A/S (NEO), Oslo, Norway) were first used to convert the original image digital number (DN) value into a radiation brightness value and extract the attitude data of the flight platform synchronized with the images. Secondly, professional hyperspectral data-processing software (PARGE, ReSe Applications LLC, Swiss) was used for geometric correction, and the geometric precision was corrected in combination with actual ground control points. Thirdly, professional hyperspectral data-processing software (ATCOR4, ReSe Applications LLC, Swiss) was used for atmospheric correction. In the end, Exelis Visual Information Solutions Inc. (ENVI) Colorado, US, software was used for image registration, and mosaic and mask were used for obtaining HySpex imaging hyperspectral data in the study area (Figure 4).

The method for obtaining the reflectance spectra of samples in this study is different from the traditional ground measurement method. The spectral information of a single sample is mixed from several rock samples. The obtained 95 position coordinates were applied to HySpex imaging hyperspectral images to obtain the reflectance spectra of the corresponding pixels, which represents the spectral reflectance of the sample.

### 2.3. Spectral Transform

The acquisition process of spectral data was affected by many factors such as instrument parameter setting and environment, which may reduce valuable information in the spectrum and increase spectral noise. Spectral transformation refers to a series of spectral transformations based on the original spectrum that is used to reduce the influencing factors, such as background environment, illumination, and atmospheric scattering on the spectrum, to highlight the characteristic spectrum of the target [39]. In the present study, the original spectrum was transformed using derivative, power, logarithm, envelope removal, and combination methods. By comparing the correlation between different transformation spectra and Cu content in the visible-near-infrared band, the optimal transformation method was selected.

Envelope removal can effectively highlight the valley and peak characteristics of the spectral curve and normalize them to the uniform spectral background, which is beneficial for extracting the characteristic bands between similar spectra [40,41]. Logarithmic transformation compresses the data without changing the nature and correlation of the data, which increases data stability but weakens the collinearity and heteroscedasticity of the model, which is conducive to reducing the influence of multiplicative factors caused by illumination changes [15,42]. Power transformation is also called gamma correction, which can expand the differences in spectrum characteristics. When gamma is greater than 1, the high-value part is generally enhanced; when the gamma is less than 1, the low-value part is enhanced [43,44]. Combinational transformation refers to the combination of two or more transformation methods that have different enhancement effects. For example, the logarithm of the reciprocal of reflectivity and the first derivative of the reciprocal of reflectivity can accurately reflect the absorption characteristics of different material components [45]. Derivative transformation can identify the minor differences between the spectral characteristics of different ground objects. The first and second-order derivatives can eliminate background noise and overlapping spectra of the resolution. The removed parts are linear or have acceptable background, terrain shadows, and noise effects on the target spectrum [45]. Higher derivatives such as fourth-order derivatives can eliminate the influence of atmospheric Rayleigh scattering [46]. For discrete spectral reflectance, the differential technique was used to calculate the spectral derivative. The formula is shown in Table 2.

### 2.4. Spectral Variable Selection

Hyperspectral data have hundreds of bands, that can detect the characteristic absorption information of detritus caused by metal elements. Due to the strong correlation between bands, information redundancy is high, which requires a long data-processing time. Bands are selected to represent the target subset of characteristic bands that retain the relatively complete useful spectral information but reduce the hyperspectral data dimension to improve the processing efficiency. In this study, three variable selection methods (continuous projection algorithm, genetic algorithm, and competitive adaptive reweighting algorithm) were used to select effective bands. The continuous projection algorithm used was a vector space collinear minimization forward variable selection algorithm, which selected a few characteristic bands that can effectively represent all bands and eliminate redundant information between the original bands. It is often used in the screening of spectral characteristic wavelengths [47]. The successive projection algorithm (SPA) method was implemented in MATLAB2014a (MathWorks.Inc, MA, Natick, USA), and the GUI_SPA toolkit was obtained from http://www.ele.ita.br/~kawakami/spa/.

The genetic algorithm was first proposed by Holland in the 1970s [48]. The algorithm is a computational model of the biological evolution process, simulating natural selection and the genetic mechanism of Darwinian biological evolution, which search for the optimal solution by simulating the natural evolution process [49]. It primarily includes the following five basic steps: (1) Generate a subset of a random variable; (2) Evaluate the fitness of individual subsets; (3) Remove the subset with low fitness; (4) Reproduce the next generation of individual subsets according to the subset with high fitness; (5) Allow mutations to form new individuals. The cycle is repeated to the second step until the condition is satisfied, at which point the optimal subset found. We used the PLS toolbox to implement the GA algorithm in MATLAB R2014a (MathWorks.Inc). The specific parameters were as follows: population size = 30, penalty slope = 0.5, window size = 1, mutation rate = 0.01, maximum iterations = 100, and replication runs = 10.

The competitive adaptive reweighting algorithm is a variable selection method with high computational efficiency based on the imitation of Darwin’s survival of the fittest. N variables’ subsets are obtained by iteration and competition from n-order Monte Carlo sampling operations. exponentially decreasing function (EDF) and adaptive reweighting sampling (ARS) methods were used in the PLS model to select variables with a large absolute regression coefficient and to remove lower-weight variables, which were then combined with 10-fold cross-validation to select the optimal subset of variables [50]. The specific steps included: (1) using Monte Carlo (MC) sampling method to randomly select 80–90% of the sample subsets in the sample set; (2) establishing the partial least squares regression (PLSR) model and calculating the weight coefficient $w_{i}$ of each wavelength; (3) using the EDF and ARS methods to remove the bands with a low-weight coefficient; (4) repeating the above steps until the conditions are satisfied. CARS was completed based on the libPLS toolbox in MATLAB R200014a.

### 2.5. Model Establishment and Accuracy Evaluation

To effectively analyze the relationship between visible-near-infrared spectroscopy and the copper content of detritus, we randomly divided the 95 detritus samples into training datasets (63 detritus samples in total) and validation datasets (32 detritus samples in total). The partial least squares regression (PLSR) model was established using spectral reflectance (independent variable) and copper content (dependent variable). PLSR has been proven to be effective and to quantify vegetation characteristics and soil element contents from remote sensing data [51]. In this study, the number of principal components was determined using the determination coefficient (*R*^2^) between the measured value and the predicted value obtained through leave one cross-validation; thus, the number of principal components corresponding to the maximum R^2^ was the optimal number of principal components.

We estimated the copper content by pixel spectral reflectance and discussed the feasibility and accuracy of estimating copper based on the hyperspectral image spectrum. Three statistical parameters were used to evaluate the performance of the model: R^2^, mean absolute error (MAE), and relative root mean squared Error (RRMSEP). The optimal model should have the highest *R*^2^ and RRMSEP, and the lowest MAE. When the value of RRMSEP is greater than 2, the model is better; the smaller the value, the worse the model performance. For the proportion of all variations of dependent variables that can be explained by independent variables through the regression relationship, the closer the value of *R*^2^ to 1, the better the model, and vice versa. The parameters are calculated as follows

(1) $$R^{2}=1-\frac{\sum_{i}^{n}{\left(Y_{i}-Y_{i}^{pre}\right)}^{2}}{\sum_{i}^{n}{\left(Y_{i}-Y_{i}^{avg}\right)}^{2}}$$

(2) $$\mathrm{MAE}=\;\frac{\sum_{i1}^{n}\left|Y_{i}-Y_{i}^{\mathrm{pre}}\right|}{n},$$

(3) $$\mathrm{RRMSEP}=\;\frac{\sum_{i}^{n}Y_{i}/n}{\sqrt{\sum_{i}^{n}{\left(Y_{i}-Y_{i}^{pre}\right)}^{2}/n}},$$

where$\;Y_{i}$, $Y_{i}^{pre}$, and $Y_{i}^{avg}$ are the true value, predicted value and average value of sample *i*, respectively; *n* is the number of samples.

### 2.6. Flowchart

**Figure 5:**
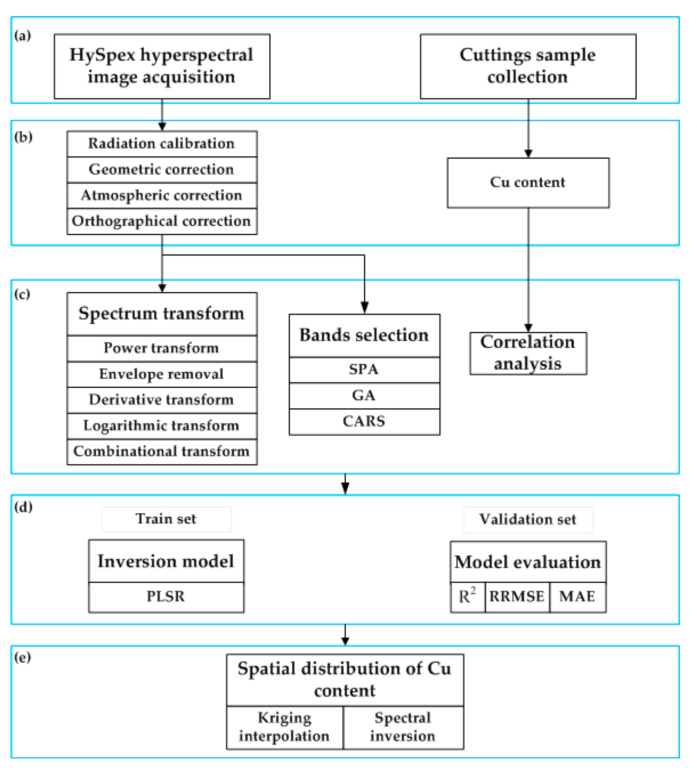
Flowchart of HySpex hyperspectral imagery inversion of Cu content: (**a**) data acquisition; (**b**) data preprocessing; (**c**) data analysis; (**d**) modeling and verification; (**e**) spatial distribution of Cu content.

The flow chart of detritus Cu content prediction is shown in Figure 5, which mainly includes the following five parts: (1) collecting HySpex hyperspectral images and detritus samples; (2) image preprocessing and Cu content measurement; (3) spectral transformation and characteristic band selection; (4) inversion model establishment and accuracy evaluation; (5) obtaining the spatial distribution map of Cu content.

## 3. Results

### 3.1. Element Content and Visible-Near-Infrared Spectral Reflectance

**Figure 6:**
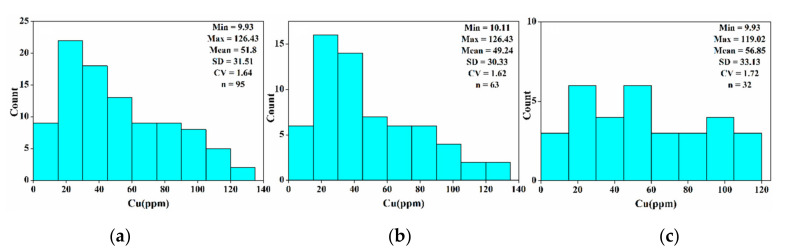
Cu element content statistical description and histograms, (**a**) all of the samples; (**b**) training samples; (**c**) verification samples; Min: minimum; Max: maximum; SD: standard deviation, CV: coefficient of variation.

To evaluate the precision and generalization ability of the constructed inversion model, we divided the data into a training set and verification set, and analyzed whether they were properly partitioned. The statistical histogram of the copper content in each sample data set is shown in Figure 6. In Figure 6a, the Cu content has a large variation in the overall sample, where the standard deviation, coefficient of variation, and the range are 31.51, 1.64 and 116.5, respectively. The data showed that the gradient of the Cu content in the samples significant changed. According to Figure 6b,c, the average, standard deviation, and coefficient of variation of the Cu content of the training sample and the verification sample were consistent with the overall samples, showing that the method of dividing the training sample and verification sample was reasonable and representative. Briefly, the analysis and modeling are representative and applicable.

**Figure 7:**
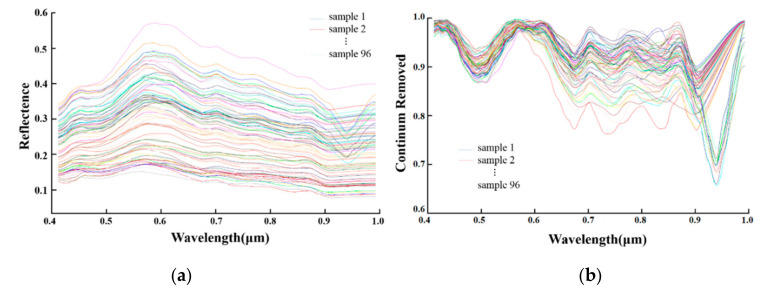
Visible-near-infrared spectral curves, (**a**) original and (**b**) envelope removal spectral curves.

Figure 7, presents examples of the reflectance spectra and envelope removal spectra. Figure 7a shows that the reflectance of the visible-near-infrared spectrum of the sample was relatively low. The spectral reflectance increased from 0.4 to 0.58 μm, then gradually decreased from 0.9 to 0.58 μm, with the reflection peak at 0.57 μm. The spectral reflectance showed two different trends in the range of 0.9 to 1.0 μm; most of the samples showed a slight uptrend within 0.02 μm, and few samples had a remarkable absorption valley at 0.94 μm.

To highlight the fine features in the visible-near-infrared spectrum, envelope removal was carried out for the original spectrum (Figure 6b). The sample spectrum showed typical absorption valleys at 0.48, 0.668, 0.752, 0.85, and 0.9 μm.

### 3.2. Correlation between Different Transform Spectra and Cu Content

When the correlation between the original spectrum and the content is not significant, the model between the original spectrum and the content cannot be established. Appropriate spectral transformation can improve the correlation between the spectrum and content. To retrieve the Cu content of the detritus using spectral data, we compared and analyzed the effects of nine spectral transformation methods.

**Figure 8:**
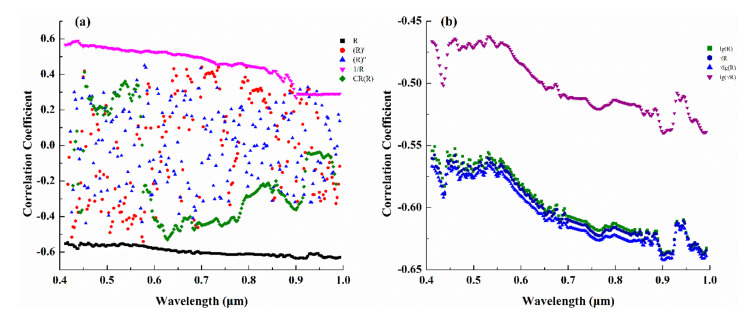
Correlation between different transform spectra and copper content: (**a**) five transformed spectra; (**b**) combined transformed spectra; R: original spectra; (R)’: first-order derivative spectra; (R)’’: second-order derivative spectra. 1/R: reciprocal spectra; CR(R): envelope removal spectra; lg(R): logarithmic spectra; $\sqrt{R}$: power spectra; $\sqrt{\mathrm{lg}\left(R\right)}$: log-power spectra; lg($\sqrt{R}$): power-log spectra.

In the visible-near-infrared band range, the correlation between the original spectrum and copper content is negative, and the absolute value of the correlation coefficient is greater than 0.545 (Figure 8a). The reciprocal spectrum is also positively correlated with copper content, with the correlation coefficient gradually decreasing to 0.267 and the correlation between Cu content and each band being lower than with the original spectrum. The correlation between derivative spectrum (first- and second-order) and envelope removal spectrum and Cu content decreased, and the correlation between each band and Cu content showed no obvious rule. The logarithmic spectrum, power spectrum, and combination transformation spectrum (log-power transformation and power-logarithm transformation) were negatively correlated with copper content, and the maximum absolute values of the correlation coefficient were 0.637, 638, 0.549, and 0.642, respectively (Figure 8b), which were all higher than the original (*r* = 0.55). The combined transform spectrum significantly increased the correlation between the bands and Cu content in the range of 420–450, 750–800, and 880–1000 nm, but also reduced the correlation between the bands and Cu content in the range of 451–700 nm.

### 3.3. PLS Model of Different Transform Spectra

**Figure 9:**
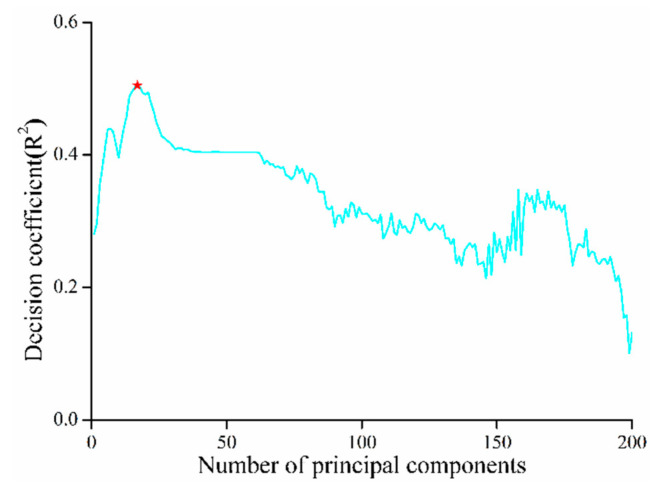
Model determination coefficient changes with the number of principal components.

In the process of constructing an inversion model using the partial least squares method, useful information is mainly concentrated in the previous principal components. Choosing an appropriate number of principal components not only enables building a model with better performance, but also simplifies the complexity of the model, thereby considerably improving modeling efficiency. The PLS model was constructed using the original spectrum, which showed the changing trend in the *R*^2^ of cross-validation with the change in the number of principal components in the model (Figure 9). Firstly, the model of R^2^ increased with the increasing number of principal components when the number of principal components was 17, and *R*^2^ reached the highest value of 0.5048. Then, it decreased with an increasing number of principal components when the number was greater than 60. The precision curve of the model demonstrated unstable oscillation. The model becames unstable with an increasing number of principal components. The main information in the spectrum was concentrated in the previous principal component, which can improve the accuracy of the model. The noise was concentrated in the later principal components, and their introduction would reduce the accuracy of the model.

From Figure 9, the best performance of the model occurred within 20 principal components. Table 3 indicates the performance of the PLS models with different transformation spectra. Based on the original spectral reflectance, the *R*^2^ of the model in the training samples and the verification samples are 0.505 and 0.486, respectively. Compared with the original reflectance spectrum, the accuracy of the model established by the reciprocal spectrum, envelope removal spectrum, first derivative spectrum and second derivative spectrum decreased, and the prediction accuracy *R*^2^ was less than 0.4. However, the accuracy of the model established by exponential and logarithmic spectra improved, in which the training accuracy *R*^2^ was 0.520 and 0.531, respectively, and the prediction accuracy *R*^2^ was around 0.490. The RRMSE and *R*^2^ of the derivative spectrum (first- and second-order), envelope removal spectrum, and derivative spectrum were all less than 2 and 0.4, respectively. The second derivative spectrum had the worst effect: RRMSEP and MAE were 0.804 and 29.705, respectively. Therefore, these transformation methods were excluded from the combined transformation process. Here, the power transformation method and logarithmic transformation method improved the performance of the model; therefore, we only combined exponential transformation and logarithmic transformation to obtain a new transformation spectrum. The combined transformation spectrum can significantly improve the prediction accuracy of the model (Table 3). The power-logarithmic transformation spectrum produced the best performance in the training and prediction sets, where the model accuracy *R*^2^ values were 0.591 and 0.586, respectively.

### 3.4. PLS Models of Different Band Selection Methods

**Figure 10:**
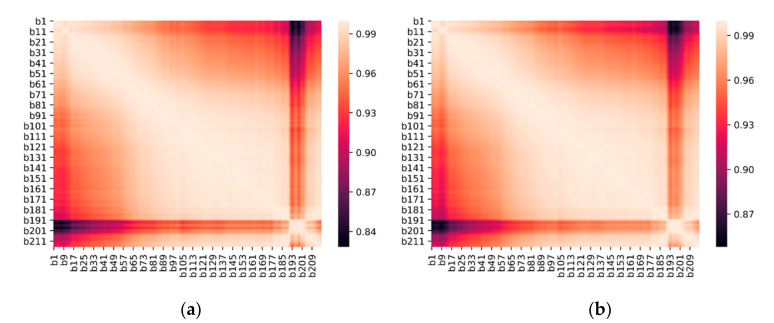
Visible-near-infrared band correlation thermal map: (**a**) original spectra; (**b**) power-logarithmic transformation spectra.

For hyperspectral remote-sensing technology, its features, such as high spectral resolution and a large number of bands, lead to a strong correlation between bands, high data redundancy, and some wavebands that are irrelevant to the element content. The map in Figure 10 shows that the correlation between the original spectrum and the combined transform spectral bands was strong, with minimum values of 0.8284 and 0.8479, respectively. When the correlation between bands was strong, using all bands to build a model will lead to overfitting.

To prove the advantages of using variable selection in hyperspectral data, we used the power-logarithmic transformation spectrum with the best model performance to discuss the influence of variable selection methods in the model. GA, SPA, and SARS were used to extract the characteristic bands of the power-logarithm spectrum, and the partial least squares model was established (Table 4). The CARS was the best-performing model in terms of spectral transformations when 20 bands were selected. The prediction determination coefficient was 0.7342; the training determination coefficient was 0.7507, which is 0.15 higher than the model prediction determination coefficient established using the full band spectrum. RRMSEP (2.21) was the largest and MAE (19.926) was the lowest among all the models of the training set. The performance of the SPA model was second best, with 42 bands, and the model’s prediction *R*^2^, RRMSEP and MAE were 0.6814, 2.175 and 21.764, respectively. The GA method was unsatisfactory, with 105 selected bands and *R*^2^, RRMSEP and MAE were 0.519, 2.102 and 23.171, respectively. The performance was close to that of the full-band model using the original spectrum.

**Figure 11:**
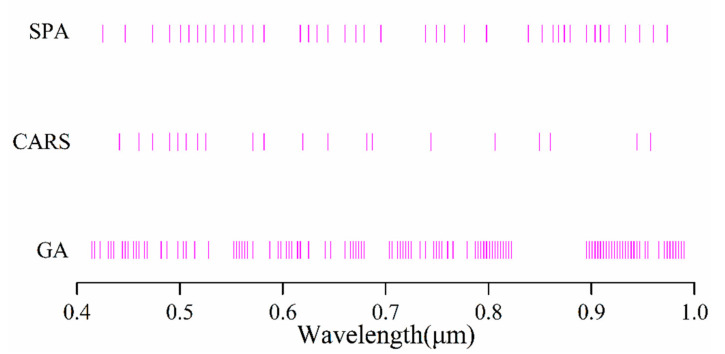
Waveband selection distribution.

Different band selection methods have different sensitivities to characteristic bands. Figure 11 shows the wavebands selected by GA, SPA, and CARS. The positions of the bands selected by CARS and SPA are relatively close. Figure 7 shows the selected wavebands have a typical correlation with the content of Cu elements, and the wavelengths including 446.7, 489.9, 516.9, 552.1, 679.1, 749.3, 798, and 903.4 nm were selected. The wavebands selected by GA were different from those selected by CARS and SPA. The poor accuracy of the model constructed based on the bands selected by the GA method was due to the significant correlation of the bands, with Cu content not being selected from wavebands with good correlation.

**Figure 12:**
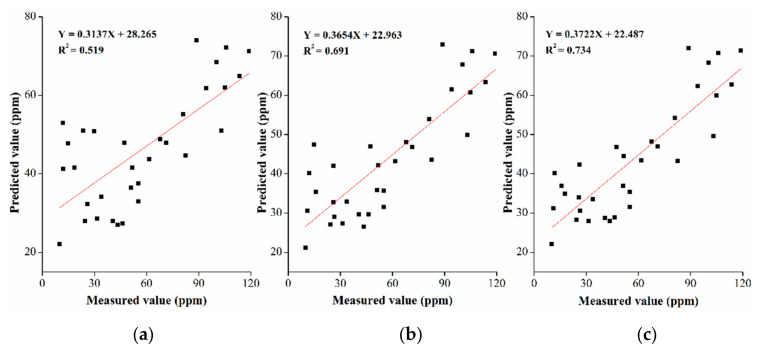
Scatter plots of observed Cu content and predicted Cu content for different band selection algorithms, (**a**) genetic algorithm (GA), (**b**) continuous protection algorithm (SPA), and (**c**) competitive weighting resampling algorithm (CARS).

SAP and CARS are better than GA in selecting the optimal bands, and the modeling accuracy of the selected band can be significantly improved. The scatter plots depict the combination of partial least squares and the observation and prediction values of Cu content in GA, SPA, and CARS (Figure 12a–c, respectively). In combination with Table 3, the data indicate that *R*^2^ of the models using SPA and CARS band selection by 0.2054 and 0.2482, respectively, and MAE decreased by 1.01 and 1.85, respectively, compared with the model established using the original full-band spectrum.

### 3.5. HySpex Imaging Hyperspectral Cu Content Extraction

**Figure 13:**
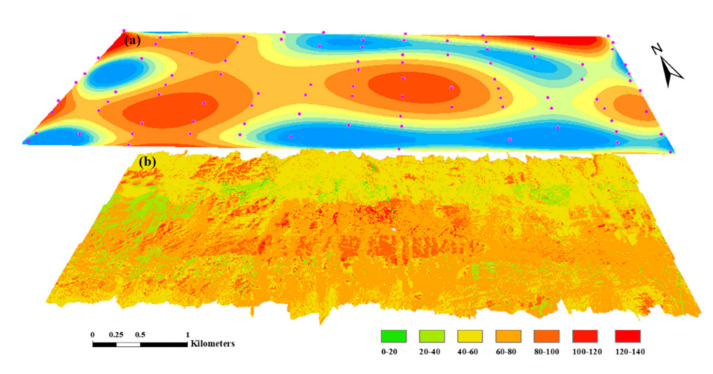
Cu content distribution map in the study area: (**a**) kriging interpolation; (**b**) hyperspectral inversion.

The spatial distribution of Cu content in the study area was obtained using the proposed method and the spatial interpolation method separately, and the inversion results of the two methods were compared and analyzed. Power-logarithm transform and band selection method were used to process HySpex hyperspectral images to obtain characteristic band hyperspectral images. The optimal model was applied to the images of the characteristic bands to obtain the Cu content distribution diagram of the study area (Figure 13b). The spatial distribution map of Cu content in the study area was obtained by kriging interpolation in the ArcGIS toolbox (Environmental Systems Research Institute, Inc, RedLands, CA, USA) (Figure 13a). Figure 12 shows that the region with higher Cu content is mainly distributed in the middle of the study area, and local high values in the upper left part. The Cu content in the study area increases first and then decreases from north to south and from west to east, and there is a high abnormal value in the middle of the study area. Both methods produced roughly the same results.

**Figure 14:**
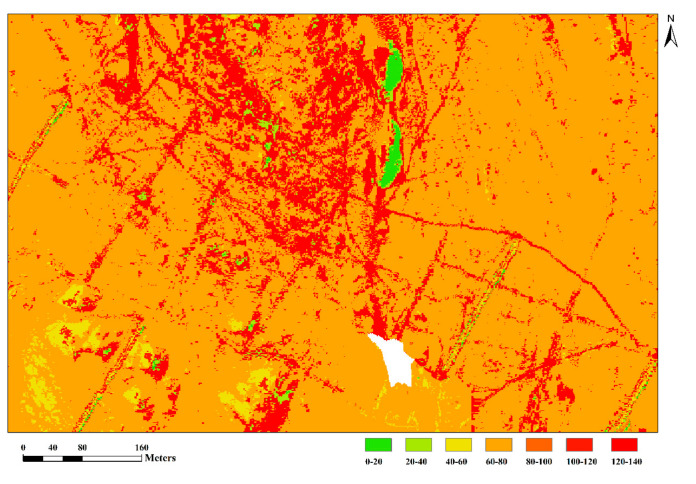
Cu content distribution map in local region.

Figure 14 depicts a partial view of the high Cu content in Figure 13a, which shows that the high Cu content is concentrated near the exploratory trench. The distribution of high abnormal Cu content in this region is consistent with the research results of the geological team, which explains the porphyry copper concealed ore bodies in this region [34]. Traditional spatial interpolation methods only obtain regional anomalies; it does not include continuous variations. Therefore, a method of estimating the content of metal elements in detritus based on the hyperspectral remote sensing pixel spectrum is proposed in this paper. The proposed method feasible and accurate, and can reduce the humanr and material resources required for geological exploration to improve the exploration efficiency.

## 4. Discussion

The use of visible-near-infrared spectroscopy to estimate element content in laboratory or field measurements has been relatively well established [52,53,54], but few studies have directly used image spectra to estimate element content. The accuracy of models directly using image pixel spectra and metal content of ground samples is not only limited by the image spatial resolution but also by the collection standards of field samples. The spatial resolution is mostly influenced by the pixel spectrum being a mixed pixel, whose spectrum represents the mixed results of various ground objects. The use of mixed-pixel spectra and single-target feature element content for modeling are not representative and applicable. Most of the previous studies used ground point spectra and point content for modeling, which were then applied the model to images. Due to the presence of mixed pixels, when the model is applied to images, the inversion accuracy is often reduced [55,56]. The HySpex image used in this article has a spatial resolution of 1m, and the corresponding ground sample is a mixed sample of 1 × 1 m (Figure 3a). The sample size was collected in a way that corresponded to the image pixel size, thus effectively reducing the influence of mixed pixels or unrepresentative objects on the model. Table 2 shows that the accuracy of Cu content estimation based on the iso-scale pixel spectrum proposed in this paper is relatively high, but its accuracy is lower than a model established in the laboratory [57].

Many previous studies have shown that the accuracy of prediction models based on the original spectral reflectance is low, encouraging researchers to use spectral processing technology to transform the original spectrum into different forms [58]. The derivative spectrum can eliminate background noise, enhance spectral features, and highlight important spectral information. The first derivative represents the slope of the reflectance spectrum, and the second derivative represents the curvature of the reflectance spectrum [59]. We found that the accuracy of the model based on the second derivative spectrum is significantly lower than that of the first derivative, which is consistent with previous research results [60,61]. The reason for this finding may be the second derivative included more prominent spectral characteristics. It is also more sensitive to noise, which reduces the quality of spectral data. In this study, although the accuracy of the derivative spectrum model enhanced the difference between the spectra, it was significantly lower than the original spectrum because the useful data or information may have been lost or the lower efficiency of the model [62].

**Figure 15:**
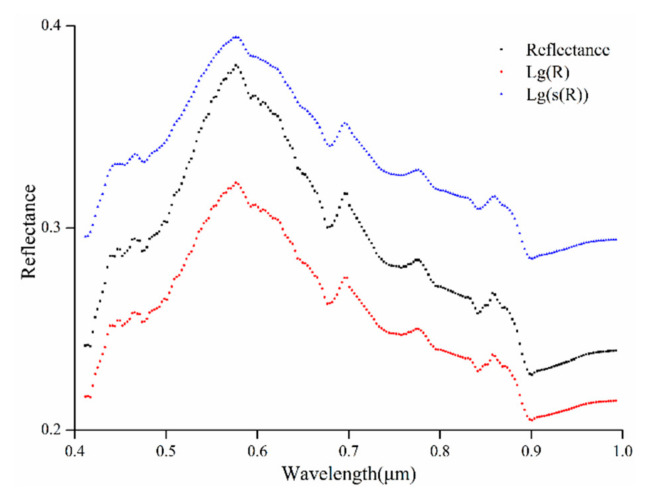
The reflectance of different spectral transforms.

Logarithmic and power transformations can remove the noise. The use of powers less than one enhances the reflectance spectrum and expands the characteristic spectrum. Logarithmic transformation can compress the data without changing the spectral properties and the low-frequency noises. Therefore, two methods were found to improve the accuracy of the model (Table 3). The logarithmic transformation can compress the data to a certain extent, reducing the influence of spectral noise in the model, so the effect is slightly stronger than that of the power transformation. Combining Figure 15 and Table 3, the accuracy of the power-logarithmic transformation model is higher than that of the single transformation method because the combined transformation may not only expand the characteristic spectrum but also eliminate the influence of noise. While producing a smoothing effect, it can improve the accuracy of the model [63].

The three waveband selection methods used in this study produced better results than the full-band spectral model in the combined transform spectrum. The best predictive performance was produced by CARS, followed by SPA and then GA. The CARS algorithm simulates the survival of the fittest principle in Darwin’s theory. In multiple iterations, the wavebands with a low weight coefficient are eliminated, leaving the characteristic bands that can represent the metal content. The spectral bands that contribute to the inversion of metal element content can also be selected when the number of selected bands is limited [64]. The GA conducts an exhaustive search for all possible band combinations, so selecting bands with typical characteristics from data with strong correlations is difficult [65]. Although the SPA algorithm selects fewer bands and simplifies the model results, the data of the SPA algorithm are somehow inaccurate. The possible explanation is the SPA algorithm mainly reduces the redundant information between bands under the condition of collinear minimization of space, which leads to the selected spectral variables only reflecting the weak correlations between spectra and the optimal subset with high information cannot be selected, thus affecting the performance of the model. In brief, it is important to select the appropriate spectral transformation and feature band-screening methods in the process of modeling.

The ranges of spectral variables selected by the three screening methods were different, but within 3nm, the spectral bands of 446.7, 489.9, 516.9, 552.1, 679.1, 749.3, 798, 870, and 903.4 nm were selected by all three methods. Due to the presence of Fe^2+^, Fe^3+^, Cu^2+^, OH, and Al-OH in iron oxides, silicates, carbonates, and chlorides, the spectral curves display relatively obvious absorption characteristics [62]. This also explains the rationality of the proposedmethod from the perspective of a physical mechanism. Notably, the SPA and CARS algorithms selected the bands of 852 and 849.3 nm, respectively, and SPA did not select the corresponding bands in this range. Previous research results indicated that the Cu characteristic response band is located at 850 nm [66]. The technical superiority of the model based on the spectral variables selected by the SPA and CARS algorithms over GA is well established. This further proves that the model proposed in this paper based on the CARS variable selection algorithm and power-logarithm transformation spectrum is more practical and has more accurate prediction ability.

## 5. Conclusions

In this study, we explored the validity of ultra-low altitude HySpex hyperspectral images for estimating the Cu content of detritus. The purpose of this method is to quickly obtain the spatial distribution of Cu content through hyperspectral images and provide technical support for the exploration of hidden minerals. The principal results indicated that:

(1) Spectral transformation technology can highlight the band that is characteristically reflected by the element content, thereby improving the predictive ability of the model;
(2) The 20 characteristic bands selected from the transform spectrum by the CARS method were input as independent variables into the PLS method to construct the detritus copper content inversion model with the highest accuracy. *R*^2^ (0.7342) was highest and MAE (19.926) was lowest in the verification set, indicating that the HySpex pixel spectrum could be used to quickly and accurately estimate the copper content in detritus;
(3) GA, CARS, and SPA can be used for quickly selecting feature bands, and the use of these feature bands for modeling can simplify the model complexity and improve prediction accuracy. CARS is the optimal feature band screening method; it reduced the complexity of the model to the greatest extent and improved the stability of the model while ensuring the accuracy of the inversion, and has a wider application prospect;
(4) Ultra-low HySpex imaging hyperspectral data have high spatial and spectral resolutions, but there were problems with information redundancy. Adopting appropriate spectral transformation technology and band selection methods to improve the prediction accuracy and data-processing efficiency can provide a low-cost and efficient method for the delineation and reduction of key mining research areas in the future.

## Figures and Tables

**Table 1:** Main parameters of HySpex imaging spectrometer.

Sensor Name	VNIR-1024	Sensor Image
Detector	SiCCD 2048 × 2048	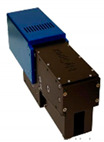
Spectral range	400–1000 nm	
Spatial pixels	1024	
Field of view angle	17°	
Extension lens	34°	
Instantaneous field of view	0.18 mrad/0.36 mrad	
Spectral sampling	2.8 nm	
Spectral number	216	
Camera weight	4.6 kg	
Camera size(cm)	31.5 × 8.4 × 13.8	
Power consumption	~6 W	

**Table 2:** Spectral transformation formulas.

Spectral Transformation	Formula
Reciprocal	$1⁄R\left(\lambda_{i}\right)$
Logarithmic	$\mathrm{lg}\left(R\left(\lambda_{i}\right)\right)$
Power	$\sqrt{R\left(\lambda_{i}\right)}$
Envelope removal	$1-E\left(R_{i}\right)$
First-order derivatives	$\frac{R\left(\lambda_{i}\right)-R\left(\lambda_{i-1}\right)}{\Delta \lambda }$
Second-order derivatives	$\frac{R\left(\lambda_{i+1}\right)-2R\left(\lambda_{i}\right)+R\left(\lambda_{i-1}\right)}{\Delta \lambda^{2}}$
Power-logarithmic	$\mathrm{lg}\left(\sqrt{R\left(\lambda_{i}\right)}\right)$
Logarithmic-power	$\sqrt{\mathrm{lg}\left(R\left(\lambda_{i}\right)\right)}$

Note: $\lambda_{i}$ is the wavelength of band i; $R^{\prime }\left(\lambda_{i}\right)$ and $R^{\prime \prime }\left(\lambda_{i}\right)$ are the first and second-order derivatives of the wavelength $\lambda_{i}$, respectively; and Δλ is the interval between two adjacent wavelengths.

**Table 3:** Partial least square (PLS) model accuracy of different transform spectra.

Spectral Transformations	Number of Principal Components	$\mathrm{Determination}\;\mathrm{Coefficient}\;\mathrm{of}\;\mathrm{Training}\;\mathrm{set}\;(R^{2})$	Validation Set		
			$R^{2}$	RRMSEP	MAE
R	17	0.5048	0.4860	2.133	22.774
(R)′	11	0.3474	0.3167	1.906	25.949
(R)″	5	0.2695	0.2612	1.761	27.755
$\mathrm{lg}\left(R\right)$	15	0.5307	0.4905	2.137	22.712
1/R	4	0.2725	0.2714	0.804	29.705
CR(R)	13	0.3834	0.3634	1.638	22.829
$\sqrt{R}$	17	0.5201	0.4901	2.127	22.690
$\mathrm{lg}\left(\sqrt{R}\right)$	17	0.5913	0.5863	2.064	21.405
$\sqrt{\mathrm{Lg}\left(R\right)}$	17	0.5869	0.5628	2.079	23.035

RRMSE is Relative Root Mean Squared Error; MAE is Mean Absolute Error.

**Table 4:** PLS model accuracy of different band selection methods.

Spectral Transformations	Number of Bands	Number of Principal Components	Determination Coefficient of Training Set (*R*^2^)	Validation Set		
				*R* ^2^	RRMSEP	MAE
GA	105	18	0.536	0.519	2.102	23.171
CARS	20	12	0.751	0.734	2.21	19.926
SPA	42	18	0.709	0.691	2.175	21.764
